# Pharmacological Significance of *Boraginaceae* with Special Insights into Shikonin and Its Potential in the Food Industry

**DOI:** 10.3390/foods13091350

**Published:** 2024-04-27

**Authors:** Shweta Gautam, Lubomír Lapčík, Barbora Lapčíková

**Affiliations:** 1 Department of Foodstuff Technology, Faculty of Technology, Tomas Bata University in Zlín, Nam. T.G. Masaryka 5555, 76001 Zlín, Czech Republic; gautam@utb.cz (S.G.); or barbora.lapcikova@upol.cz (B.L.); 2 Department of Physical Chemistry, Faculty of Science, Palacky University in Olomouc, 17. Listopadu 12, 77146 Olomouc, Czech Republic

**Keywords:** shikonin, acetylshikonin, *Boraginaceae*, naphthoquinone

## Abstract

Shikonin is a naphthoquinone pigment present in the hairy roots of the plant species from the *Boraginaceae* family. The compound has been well investigated for its highly efficient medicinal, antioxidant, and antimicrobial properties. Various extraction methodologies have been employed to maximise yield while minimising waste production of shikonin and its derivatives. Despite substantial research on shikonin and Boraginaceae plants, a research gap persists in the food industry and extraction technologies. This review addresses crucial aspects of shikonin deserving of further exploration. It begins by elucidating the attributes of the *Boraginaceae* plants and their medicinal traits in folklore. It proceeds to focus on the roots of the plant and its medicinal properties, followed by extraction procedures explored in the last fifteen years, emphasising the novel technologies that have been chosen to improve the yield extract while minimising extraction times. Furthermore, this review briefly outlines studies employing cell culture techniques to enhance in vitro shikonin production. Lastly, attention is directed towards research in the food industry, particularly on shikonin-loaded biodegradable films and the antioxidant activity of shikonin. This review concludes by summarising the future potential in food science and prominent research gaps in this field.

## 1. Introduction

Shikonin and its derivatives are naphthoquinone, bright-red-purple pigments found in the *Boraginaceae* family that have been the subject of intensive research owing to their remarkable medicinal attributes. These compounds exhibit potent antibacterial and antioxidant properties and have shown promise in addressing various health conditions, making them useful for pharmaceutical applications [1]. Researchers have explored their potential in treating different types of cancer and wound-healing properties [2]. Within the vast *Boraginaceae* family, numerous species have been identified and studied for their shikonin content, including *Echium*, *Alkanna*, *Arnebia*, *Lithospermum*, *Onosma*, *Buglossoides*, *Craniospermum*, *Cynoglossum*, *Nonea*, *Stenosolenium*, *Eritricium*, and *Anchusa* [3,4], where investigations into the specific characteristics and variations in shikonin composition have provided valuable insights. The study of these genera has expanded the knowledge of shikonin pigments and contributed to a broader understanding of the Boraginaceae family’s ecological, physiological, and pharmacological aspects [5]. As research in this field continues to evolve, exploring shikonin and its derivatives in medicinal applications and ecological interactions remains an exciting avenue. The *Boraginaceae* family’s multifaceted nature and its constituents’ therapeutic potential position these plants as valuable resources for pharmaceutical and ecological studies.

The *Boraginaceae* family consists of herbs, shrubs, and, rarely, trees. The flowers of the plant are known to be bisexual, whereas the fruit is a drupe, capsule, or schizocarp, and the seeds are endospermous. Geographically, the *Boraginaceae* family’s wide distribution features its adaptability to various climates and ecosystems. From the landscapes of Asia to the temperate regions of Central Europe and North America, these plants have established a global presence [6,7]. The annual flowering season occurs from June to September in temperate areas, aligning with entomophilous bees’ activity [8]. Certain species within the *Boraginaceae* family have long been utilised in various regions, including China, India, and Nepal, as natural food colourants and textile dyes and have a rich history of medicinal use [6,9]. However, not all the species in *Boraginaceae* are known for their therapeutic properties and the presence of shikonin. Some species are also considered toxic. The borage plant, also known as the “forget-me-not”, and the genus *Cynoglossum* are examples that are considered problematic weeds due to the serious illnesses they can cause upon consumption. The toxicity associated with these plants is attributed to the presence of potent pyrrolizidine alkaloids (lycopsamine, supinidine, amabiline, intermedine), which have been identified as carcinogenic, mutagenic, and hepatotoxic [10,11,12]. These compounds can interact with cellular functions, induce abnormal mitosis, and contribute to tissue necrosis [13,14].

While some genera have been reported to be highly toxic, other genera within the *Boraginaceae* family have been associated with therapeutic properties. For instance, *Lithospermum*, *Erythrorhizon*, and *Arnebia guttata* are known for their cooling effects [15]. This cooling property has proven beneficial in managing conditions like fever. The naphthoquinone pigments play a key role in lowering body temperature, making them valuable remedies for ailments such as measles, chickenpox, and typhus-induced high fever [15]. The recommended dosage typically falls between 3 and 10 mg, with 3 mg considered standard [16]. 

The past decade has seen an immense increase in the manufacture of edible films, coatings, and other active and intelligent packaging materials developed for specific foods to enhance the shelf life and simultaneously provide food-contact-based monitoring of freshness in real time [17]. Such packaging materials are based primarily on the reaction in the packaging material due to chemical changes in the food during storage. Based on our previous patent on paper-based composite planar material, developed as an intelligent packaging material for microwave applications, the presented review holds the innovative aspect for the development of similar intelligent surface coatings sensitive to colour change upon a change in the pH of the food products [18]. The increased research and demand for such packaging materials dictate the necessity of the presented review. The detailed and updated information on the extraction methodologies and existing trends of shikonin in food preservation, packaging, freshness, and pH indicators would act as the base of future trends in food technology. In light of this, this review begins by briefly covering the pharmacological properties of the aerial plant part and its usage in folklore medicine. It then extensively discusses the roots of Boraginaceae species, including their medicinal properties, in situ shikonin production, antioxidant activity, and toxicity. 

## 2. Use of Aerial Plant Parts

The roots from the *Boraginaceae* have been predominantly investigated in scientific research; however, the awareness of the medicinal attributes of aerial plant components, such as leaves, shoots, and flowers, has not been entirely excluded. The *Boraginaceae* family enjoys popularity in traditional medicine folklore. Among the aerial plant parts, the leaves of *Echium* species have been studied extensively and documented in Roman literature for their mood-enhancing properties when subjected to boiling with wine [19]. In Turkey, the leaves of the same species are recognised as a mild remedy for rheumatic pain and abscesses, while seed oil is proposed as a potential dietetic supplement. In Iran, the flower petals are acknowledged for their efficacy in treating the common cold and pneumonia and as a sedative [20]. The aerial part is recognised in Italy for its diuretic properties [21]. In Germany, leaves and flowers are cited for their efficacy in treating bruises and as a diuretic [22]. The leaf decoction of the *Cordia* species has been reported to exhibit therapeutic effects on liver ailments and fever in North and South America [23]. In some areas of India, stem bark is employed to treat jaundice, and the entire plant serves as a snakebite remedy [24]. The work by Jin et al. 2020 dictates the ethnomedicinal uses of the Echium species from around the world, emphasising the antioxidant activity and constituents of plant parts [25]. 

While the efficacy of folklore medicine for the genus *Boraginaceae* is notable, a comprehensive understanding of its therapeutic effects needs to include medical and chemical elucidation as per the observations in the literature. In a recent investigation, 17 species within the *Boraginaceae* family were subjected to study for their secondary metabolites through the utilisation of high-performance capillary electrophoresis (HPCE) and multivariate chemometric techniques. The analysis unveiled the presence of various compounds, including but not limited to allantoin, rosmarinic acid, and chlorogenic acid (Table 1). The shoots of the examined species were identified as reservoirs of six distinct compounds: rosmarinic acid, chlorogenic acid, p-hydroxybenzoic acid (PHBA), rutin, allantoin, and hydrocaffeic acid. In contrast, the roots were found to contain four specific compounds: rosmarinic acid, allantoin, hydrocaffeic acid, and shikonin. The concentrations of these compounds exhibited variations among the different species and are responsible for the therapeutic properties [26].

## 3. Exploring Shikonin: Key Bioactive Compound in *Boraginaceae* Plant Roots

### 3.1. Shikonin Research in Medicine 

The root represents the most valuable anatomical component of the *Boraginaceae* plant family. It has been subjected to extensive scientific scrutiny spanning a period exceeding two decades owing to the naphthoquinone pigment present in the rhizome [27]. Detailed research works exist in the medical field, especially in Traditional Chinese Medicine [28]. Shikonin’s medicinal properties and therapeutic effects have motivated researchers to investigate the molecular pathways and mechanisms associated with various pathological conditions, elucidating its role as a potential remedy. This investigation has resulted in the successful discovery of shikonin’s anticancer properties across a spectrum of malignancies, including gastric, cervical, prostate, cutaneous, leukaemia, colorectal, pulmonary, and mammary cancers [29,30,31]. 

Research exemplified by Kim et al. 2017 has supported shikonin’s anticancer attributes by investigating cancer’s critical characteristics, namely, cellular proliferation, migration, invasion, and programmed cell death [32]. Their findings also reported the induced autophagy in cancer cells. Furthermore, it was established that shikonin exerts inhibitory effects on specific genes responsible for regulating glycolytic processes, thereby contributing to its anticancer mechanisms. Tumour cells rely on the energy generated through glycolysis. Hence, the inhibition of glycolysis-inducing genes leads to the suppression of aerobic glycolysis, subsequently hampering the growth of human cancer [33,34,35,36,37,38,39]. Shikonin has been identified as a compound that interferes with glycolysis while also binding to specific cell growth pathways, inhibiting both cancer cells and normal cells during inflammatory processes [40,41,42,43]. The efficacy of shikonin against various cancers, including leukaemia, gallbladder, breast, stomach, and acute promyelocytic cancer, can be attributed to its dual action. This involves simultaneously inhibiting genes associated with apoptotic cell death and activating genes responsible for stress-induced apoptosis [43,44,45,46,47,48,49,50,51,52]. 

In addition to the documented anticancer effects attributed to shikonin, numerous scientific investigations, particularly within the Chinese Medical community, have investigated multiple pathways through which the compound exerts its anticancer properties in human and murine models [53]. Shikonin exhibits distinct interactions with various cellular proteins, depicting diverse cellular responses. Within mitochondria, shikonin induces the dissipation of mitochondrial membrane potential, culminating in apoptotic cell death through mitochondrial pathways [54,55,56,57,58]. It heightens endoplasmic reticulum stress concurrently, contributing to apoptotic cell death via this alternate cellular route [59]. Additionally, shikonin prompts necrosis-dependent cancer cell death by upregulating the necrosis factor receptor-interacting protein [60,61,62,63,64]. Beyond its anticancer efficacy, shikonin’s derivative, alkannin, also demonstrates analogous activities against cancer and inflammation [65,66,67]. Additionally, shikonin also shows the following activities: anti-ischemic, anti-inflammatory (particularly in cardiovascular diseases and rheumatoid arthritis), antibacterial, antiviral, antiacne, antiulcer, wound healing, and scar healing, as well as therapeutic intervention for endometriosis, lowering the body temperature, detoxification, and as remedies for ailments such as measles, carbuncles, burns, macular eruptions, hepatitis, vaginitis, cervitis, empyrosis, pathopyretic ulcer, and purpura eczema [30,31,68,69,70,71,72,73,74,75,76,77,78,79,80,81,82]. This expansive array of therapeutic attributes highlights the potential of shikonin and its derivatives as versatile agents with diverse applications in oncology, inflammation, and various other pathological conditions [44,45,83].

### 3.2. Extraction Methods of Shikonin 

Shikonin and alkannin are enantiomeric compounds. These compounds, along with their derivatives, namely, deoxyshikonin, diacetylshikonin, 1-methoxy acetylshikonin, β,β′-dimethylacrylshikonin, 2,3-teracrylshikonin, β-hydroxyisovalerylshikonin (β-HIVS), β,β-dimethylacrylalkannin, acetylalkannin, β-acetoxy-isovalerylalkannin, and β-hydroxyisovalerylalkannin, are also classified as naphthoquinones [84]. Extraction and isolation methodologies for these compounds have been well documented in the scientific literature over the past two decades. Table 2 summarises the various response surface methodology-optimised extraction procedures reported in the literature from the last fifteen years. The investigation and isolation of these compounds, including their derivatives, contribute to understanding their chemical properties and potential applications in the pharmaceutical and food industries. 

Since naphthoquinone pigments are sensitive to oxidative, thermal, and photodegradation, the selection and optimisation of extraction time, temperature, and solvent are crucial. According to Xie et al. 1997 [85], shikonin and its derivatives begin to degrade at temperatures exceeding 60 °C. Compared to longer hours of Soxhlet extraction, ultrasound-assisted extraction (UAE) is more efficient in rapidly extracting below the degradation temperature. Extractions at 35 °C in methanol, 85% ethanol and hexane, 38.6 °C in ethanol, and 51.5 °C in 72% ethanol have been reported as optimal temperatures for UAE [84,86,87,88]. However, for supercritical CO_2_ extraction, a higher temperature has been reported to be more efficient [89,90]. According to the works of Akgun et al. 2017 [89], the highest yield for alkannin was obtained at 80 °C due to the efficient diffusivity of CO_2_ with increasing temperatures. Another study used a much simpler extraction approach, employing freeze drying of the roots in liquid nitrogen and grinding and dispersion in hexane. The risk for thermal degradation was avoided by this methodology while resulting in a yield of 800 mg shikonin from 40 g of sample [91]. 

**Table 2:** Summary of optimised extraction methodologies of shikonin and its derivatives with the type of solvent used in the past fifteen years.

Plant Species	Extraction Method	Compounds Extracted and Yield	Solvent Used	Reference
*Arnebia euchroma*	UAE: solid/liquid ratio = 11.27:1; ultrasonic time = 86.98 min; extraction temperature = 38.67 °C; ultrasonic power = 92.87 W.	Shikonin: 1.26%	Ethanol (95%)	[84]
*Arnebia euchroma*	UAE: solid/liquid ratio = 1:12; ultrasonic time = 35 min; extraction temperature = 35 °C; surfactant concentration: 0.0004 g/L; ultrasonic power = n/a.	Shikonin: 30.64 mg/L	Methanol	[86]
*Arnebia euchroma*	Ionic liquid–UAE: ultrasonic time = 5 min; ionic liquid = 150 µL with 50 mg sample powder; ultrasonic power = 100 W.	Shikonin: 0.35 µg/mg β,β′ dimethylacrylshikonin: 2.21 µg/mg	Ionic liquid: [C6MIM][BF4]	[82]
*Lithospermum erythrorhizon*	Traditional extraction using freezing and grinding 40 g root powder in liquid nitrogen. Multiple washes in the solvent to concentrate the compound followed by complicated isolation by high-performance liquid chromatography (HPLC).	Shikonin: 800 mg	Hexane	[91]
*Alkanna tinctoria* (L.) TAUSCH subsp. *subleiocarpa*	Supercritical CO_2_ extraction at 80 °C, 175 bar pressure, and 5 g/min CO_2_ flow rate.	Alkannin: 1.47%	Supercritical CO_2_	[89]
*Arnebia euchroma* (Royle) Johnston *Lithospermum erythrorhizon* *Arnebia guttata*	Continuous sampling dynamic microwave-assisted extraction coupled with online HPLC for extraction, separation, and determination: sample weight: 100 mg; particle size: 110 mesh; extraction time: 9 min; solid/liquid ratio = 1:100 (g/mL); flow rate: 1.65 mL/min; microwave power = 240 W.	Shikonin: 0.66–0.92 µg/mg β,β′ dimethylacrylshikonin: 3.02-8.88 µg/mg	80% ethanol	[92]
*Arnebia euchroma*	Surfactant-assisted UAE: surfactants used: Tween 20; solid/liquid ratio= 1:12 g/mL; extraction time: 35 min; extraction temperature: 35 °C.	Shikonin: 30.64 mg/L	Hexane	[86]
*Onosma hookeri Clarke*. var. longiforum Duthie	UAE: solid/liquid ratio = 1:37 g/mL; extraction time: 77.2 min; extraction temperature: 51.5 °C; ultrasonic power = n/a.	Isovaleryl shikonin: 0.094% Tigloylshikonin: 0.223%	72.4% ethanol	[87]
*Arnebia euchroma*	Homogenate extraction: solid/liquid ratio = 1:10.3 g/mL; extraction time: 4.2 min.	Shikonin: 97 µg	78% ethanol	[93]
*Onosma visianii*	Soxhlet extraction: extraction time: 5 h; sample amount: 80 g root powder in 500 mL solvent. The crude extract obtained was then subjected to proton nuclear magnetic resonance (H-NMR) for quantification.	Isovalerylshikonin: 37.2 mg Isobutyrylshikonin: 63.2 mg Acetylshikonin: 16.6 mg Hydroxyisovalerylshikonin: 2.4 mg Shikonin-β,β-dimethylacrylate: 58.7 mg Propionylshikonin: 1.1 mg 5,8 dimethoxy acetylshikonin: 8.1 mg 5,8 dimethoxy isobutyrylshikonin: 0.6 mg 5,8-*O*-dimethyldeoxyshikonin: 4 mg	Hexane	[94]
*Arnebia euchroma*	Matrix solid-phase dispersion: dispersant: C18; solid/liquid ratio = 1:3 g/mL.	Shikonin Acetylshikonin Isobutylshikonin β, β-dimethylacrylshikonin Isovalerylshikonin	Ethanol	[95]
*A. tinctoria; Alkanna tinctoria subsp. Subleiocarpa*, *A. tubulosa; Alkanna tubulosa*, *A. anatolica; Alkanna tinctoria subsp. Anatolica*, *A. hirsutissima; Alkanna hirsutissima*	Supercritical CO_2_ extraction at 80 °C; 175 bar pressure and 5g/min CO_2_ flow rate; solvent/feed ratio = 30.	Alkannin: 1.47%	Supercritical CO_2_	[90]
*Arnebia euchroma* (Royle) Johnst	Ultrasound-assisted ionic liquid solid–liquid extraction coupled with aqueous two-phase extraction: ionic liquid used: 440 µL [C4MIM][BF4]; 0.14 g sodium dodecyl benzene sulfonate; ultrasound time = 10 min; ultrasound power: 120 W; pH 7; centrifugation at 2000 rpm for 5 min.	Shikonin: 0.88 mg/g Acetylshikonin: 2 mg/g β, β-dimethylacrylshikonin: 5.43 mg/g	Acetonitrile	[96]
*Onosma glomeratum*	UAE: solid/liquid ratio = 1:40 g/mL; extraction time: 30 min; extraction temperature: 30 °C.	Naphthoquinones: 0.98%	85% ethanol	[88]

n/a: Data not available.

The literature consistently identifies UAE as the predominant method for the extraction of shikonin. In addition to the conventional UAE, Xiao et al. 2011 [82] introduced a modified approach by incorporating an ionic liquid (IL). Ionic liquids are particularly advantageous due to their broad applicability in synthesis, catalysis, separation, and electrochemistry [82]. They possess negligible vapour pressure, high chemical and thermal stability, and good solubility in organic and inorganic compounds, rendering them environmentally friendly or green solvents [97,98]. In their study, the authors conducted conventional UAE and Soxhlet extraction and modified UAE with an ionic liquid for comparative analysis. No significant differences were observed in the extraction yields among the three methods, emphasising the potential utility and equivalency of the ionic liquid-enhanced approach despite the inherent challenges posed by shikonin’s thermal instability and susceptibility to oxidation [28,36]. The advantage of the modified UAE method with IL, as introduced by Xiao et al. 2011 [82], was the reduced solvent consumption and accelerated extraction time of 5 min, in stark contrast to the 30 min required for conventional UAE and the 300 min for Soxhlet extraction. A parallel investigation conducted by Sun et al. 2019 [96] incorporated an additional centrifugation step to establish a liquid–liquid–solid three-phase system. The study included conventional UAE and heat reflux extraction (HRE) for comparative purposes, utilising 400 µL of [C4MIM][BF4] as the IL. Results indicated that the proposed method exhibited higher extract yields than HRE but lower yields than conventional UAE. The key advantages of this approach were, once again, the minimised solvent consumption and reduced extraction time. A distinct approach proposed by Cui 2014 [86] introduced a surfactant to the powdered root during extraction. The determination of the critical micellar concentration (CMC) of the samples, followed by extraction, revealed that the presence of the surfactant, particularly under the CMC, enhanced the extraction yield. This innovative method presents a niche worth exploring, leveraging surfactant-induced extraction efficiency improvements. 

Molecular imprinting is a specialised technique designed to create active binding sites with specific shapes and sizes for the target molecule [99]. This template-based methodology offers a distinct advantage in selectively isolating the target compound from complex mixtures [100]. Tsermentseli et al. 2013 [100] employed methacrylic acid (MAA) and 2-dimethylaminoethyl acrylate (DEAEMA) as the selected polymers for creating an imprinted polymer alongside shikonin from hexane extracts of *Alkanna tinctoria* roots (with a shikonin/MAA/DEAEMA ratio of 1:4:20). The resulting particle complex underwent solid-phase extraction. It was found that up to 72% of recovery was achieved from the hexane extracts of shikonin, commercial shikonin sample, and commercial shikonin pharmaceutical ointment.

### 3.3. In Situ Production of Naphthoquinones

In addition to the extraction, isolation, and characterisation of shikonin, considerable attention has also been directed towards Boraginaceae species in cell culture. The medicinal applications of the compound have prompted researchers to explore methodologies for augmenting its production within laboratory settings. A study conducted by Fu et al. 1999 [101] investigated the impact of fungal elicitors prepared from *A. niger* and *R. oryzae* on shikonin production in suspension cultures of *Arnebia euchroma*. The results indicated that introducing fungal elicitor *A. niger* to the culture medium yielded 61.62 mg/L shikonin after 8 days of culture incubation, which was “1.54-fold” higher than the control samples, which yielded a maximum of 39.98 mg/L shikonin extract after 12 days’ culture. Alternatively, the elicitor from *R. oryzae* boosted shikonin production to 76.87 mg/L, “1.92 times” higher than the control. The study also investigated the effect of the time of addition of fungal elicitors to the culture on the shikonin extract yield. It was found that the shikonin production was maximum to a concentration of 89.75 mg/L when the *R. oryzae* elicitor was added on the sixth day of the culture growth. The findings also established a synergistic effect between the fungal elicitors and *n*-hexadecane, yielding a concentration of 245.68 mg/L of shikonin [101]. An investigation to study the influence of ethylene on the biosynthesis of shikonin derivatives in shoot cultures of *Lithospermum erythrorhizon* showed that shoots cultured in nonventilated Petri dishes exhibited a higher production of shikonin derivatives compared to those cultured in well-ventilated Petri dishes [102]. The authors, Touno et al. 2005, speculated that the presence of ethylene in the shoots was the main reason for shikonin production, and a nonventilated Petri dish generated more ethylene [102]. Another study, conducted on shoot cultures of *Lithospermum erythrorhizon* in flasks coupled with in situ extraction using *n*-hexadecane, demonstrated that shikonin concentration in flask cultures was 106.2 mg/L, which was three-fold higher with extraction as opposed to the control sample (42 mg/L). In a bubble column bioreactor, a shikonin yield of 572.6 mg/L and a dry cell mass of 15.6 g/L were obtained after 54 days, with shikonin being consistently produced at a rate of 10.6 mg/L per day [103]. A study by Kim et al. 1990 [104] discussed increased shikonin production through cell immobilisation in calcium alginate beads and adding *n*-hexadecane to the cell cultures. The findings suggest that conducting in situ extraction at an earlier developmental stage and employing lower concentrations of beads resulted in a “7.4-times” increased shikonin production compared to samples without *n*-hexadecane. The study emphasised the significance of dissolved oxygen concentration in shikonin production and proposed that employing a bioreactor system incorporating immobilised cells and in situ extraction and ensuring an adequate oxygen supply could represent an efficient approach for shikonin production [104].

### 3.4. Antioxidant Activity of Shikonin

**Figure 1:**
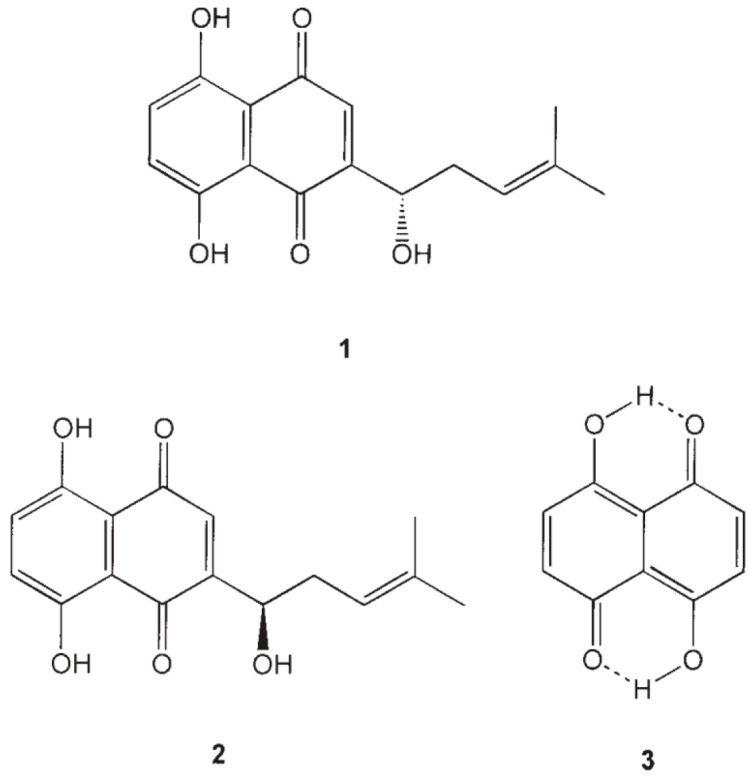
Structure of alkannin (**1**), shikonin (**2**), and naphthazarin (**3**).

Alkannin and shikonin are chiral compounds and are hydroxyl derivatives of naphthazarin (Figure 1) [105]. Their antioxidant activity is due to the component naphthazarin and alcohol hydroxyl and alkenyl side chain [106]. According to the works of Kourounakis et al. 2005 [107], the compounds alkannin, shikonin, and naphthazarin inhibited lipid peroxidation at a concentration ranging from 45 to 100 µM. The highest half-maximal inhibitory concentration (IC_50_) value after 45 min of activity was obtained for alkannin (45 µM), followed by shikonin (28 µM) and naphthazarin (20 µM). 

Another similar study was conducted on alkannin and shikonin (pure component, monomeric, and polymeric forms) as potential antioxidant agents for lard, sunflower, olive, and corn oil [108]. The antioxidant activities (performed as peroxide value) of compounds were studied against caffeic acid for comparison. For lard, the 0.04% *w*/*w* extract showed the highest antioxidant activity, whereas the 0.02% *w*/*w* dichloromethane extracts of the compounds with 0.02% *w*/*w* caffeic acid showed a synergistic effect. For other oil samples, the following extracts were found to be the most effective: olive oil: 0.02% *w*/*w* extract of monomeric shikonin; corn oil: 0.05% *w*/*w* extract of monomeric alkannin; and sunflower oil: 0.02% *w*/*w* extract of shikonin + 0.02% *w*/*w* citric acid [108]. The antioxidant activity of shikonin and its derivatives, namely, naphthazarin, deoxyshikonin, alkannin, shikonin, acetylshikonin, isovalerylshikonin, and β,β-dimethylacrylshikonin, has been reported to be highly influenced by the stearic hindrance between the derivatives and the accessibility of the branched iso-hexenylnaphthazarins to the radical site [109,110]. It has been reported that the parent compound, naphthazarin, has the highest antioxidant activity, followed by acetylshikonin, alkannin, shikonin, deoxyshikonin, isovalerylshikonin, and β,β-dimethylacrylshikonin [109]. Furthermore, the number of available phenolic groups capable of scavenging the free radicals is the same in all the derivatives [111]. Therefore, the difference in their activity could be because of their side chain and purity percentage [109,111].

### 3.5. Toxicity

Most research affirms shikonin exhibits “little to no” toxicity [112,113]. Nevertheless, while corroborating this assertion, an exhaustive literature review conducted by Yadav et al. 2022 [29] identified a limited number of studies indicating minor toxicity of shikonin in rat models. Furthermore, the review article highlighted notable variability, such as the observed variation in toxicity between in vitro and in vivo studies. Reduced toxicity was noted in in vivo studies compared to their in vitro counterparts. The study also described the distinctions in the bioavailability of shikonin when administered orally versus intraperitoneally or intravenously, which might account for the perceived “little to no” toxicity upon oral administration. Furthermore, in vitro studies by Figat et al. 2021 and Cheng et al. 2019 indicated that shikonin has higher cytotoxicity than acetylshikonin against the liver tissues of humans and rats, but acetylshikonin causes antigen toxicity against cyclophosphamide-induced genotoxicity [114,115]. According to the authors, the higher toxicity could be due to the difference in the absorption rate, environment, exposure time, and metabolism rate. Overall, the toxicity of shikonin and its derivatives depends on the mode of dosage (intraperitoneal, intravenous, or orally), with oral dosage being the least toxic [29]. 

## 4. Use of Shikonin in the Food Industry

Shikonin and its derivatives have been investigated in food science and technology due to their antibacterial and antioxidant activities and halochromicity. Researchers such as Li et al. 2021 [116] and Wan et al. 2021 [117] have reported on the potential efficacy of shikonin against *Listeria monocytogenes* and *Staphylococcus aureus* biofilms, respectively. Investigations involved assessing shikonin’s minimum inhibition concentration (MIC) against the respective microbial species, revealing MIC values of 25–100 µg/mL for *L. monocytogenes* and 35–70 µg/mL for *S. aureus*. Shikonin exhibited greater effectiveness against *S. aureus* compared to natural extracts of punicalagin (from pomegranate peel) [118], chlorogenic acid [27], and resveratrol [83]. Additionally, shikonin was found to diminish the virulence gene expression of *S. aureus* while successfully inhibiting biofilm formation. In a study conducted by Wan et al. 2021 [117], shikonin solution was applied to food models, namely, beef, seaweed, and mulberry juice, at a concentration of 35 µg/mL for 5 min for seaweed and beef samples (dipped in shikonin solution and 5 mL added to mulberry juice to make a final concentration of 35 µg/mL). The samples were then added with a bacterial suspension of *S. aureus* to make the concentration of 10^5^ CFU/mL or 10^5^ CFU/g, followed by storage at 25 °C for 24 h. It was found that the number of colonies dropped to 3.08 log CFU/mL compared to the untreated sample value of 6.45 log CFU/mL for seaweed, 2.86 log CFU/mL compared to the untreated sample value of 6.62 log CFU/mL for beef, and to 2 log CFU/mL compared to an untreated sample value of 7.7 log CFU/mL for mulberry juice. The effect was observed to be the best in mulberry juice, with no significant effect on the overall appearance of the food models [117]. Subramaniam et al. 2015 [119] conducted a study with a parallel objective of prolonging the shelf life of both pasteurised and unpasteurised fruit juices. Acetylshikonin and β, β dimethylacryl shikonin extracted from *Arnebia nobilis* were combined with nisin (250 ppm) in a 1:1 ratio and evaluated against eleven pathogenic microorganisms, including *S. aureus*, *L. monocytogenes*, and *B. cereus*. The compounds demonstrated efficacy against all Gram-positive bacteria over 9 storage days but exhibited ineffectiveness against Gram-negative bacteria. As per the work of Lee et al. 2015 [120], the ineffectivity of the shikonin derivatives could be due to their affinity to peptidoglycan of cytoplasmic membranes and the activity of ATP-binding cassette (ABC) transporters (regulating the import and export of substances across plasma membranes) [121]. These investigations contribute valuable insights into the potential utilisation of shikonin as a natural food preservation agent, particularly effective against a diverse spectrum of microorganisms.

Furthermore, predominant research in the field of food science pertaining to shikonin has been centred on the formulation of halochromatic films designed to investigate colour alterations indicative of food spoilage, particularly in fresh meat and seafood [122,123,124,125,126]. Table 3 summarises relevant information, including the plant species employed, base materials utilised, concentrations applied, and the corresponding applications in food studies. 

Shikonin-loaded indicator films have been developed in two ways: by directly adding the compound to the polymer solution and by immersing the films in the ethanolic suspension of shikonin. According to the works of Swarup et al. [126], a 10 wt% ethanolic solution of shikonin provides the best colour-indicating properties, whereas >10 wt% reduced the mechanical properties of the films. Furthermore, the best antioxidant property (determined by DPPH and ABTS) was found for the gelatin–cellulose nanofibre films (CNF). According to the authors, this “exceptionally high” antioxidant might have been due to the swelling ratio of gelatin–CNF films, which influenced the interaction of shikonin with free radicals. It can be suggested that the activity of shikonin as an effective antioxidant film highly depends on the quantity, type of shikonin sample (pure component, powdered root extract, or ethanolic extract), type of polymer, and swelling ratio [112,128,131].

## 5. Conclusions

*Boraginaceae* is an extensively studied genus with highly potent plant species. While the aerial component of the *Boraginaceae* species has been used for treating illnesses worldwide, their root has received the most attention. The naphthoquinone pigment, shikonin, and its derivatives in the root are highly potent compounds capable of treating diseases, including different forms and types of cancers and skin conditions such as eczema, acne, wounds, and scars. Alkannin and shikonin have been studied thoroughly in medicine and pharmacy to understand their action mechanisms against several diseases, especially their efficacy towards cancer cells. There was provided in this review article the insight into the extraction methodologies employed for the extraction of shikonin. Research on UAE and its modification with ionic liquids holds the advantage of having less extraction time and temperature and low solvent consumption compared to conventional Soxhlet extraction. Furthermore, its extraction can be enhanced by employing in situ cell culture using fungal elicitors or enhancing the growth environment using bubble column reactors and cell immobilisation by calcium alginate beads. Since shikonin and its derivatives possess “little to no” toxicity and have substantial antioxidant activity, they are highly suitable for utilisation in the food industry. However, there is a considerable literature gap in studies focused on green technologies for extraction of the compound and its use in food formulations to expand the practicality of the compound for consumers. 

## Figures and Tables

**Table 1:** Summary of the detected secondary metabolites as per the study conducted by Dresler et al. 2017 [26].

Plant Species	Compounds Detected	
	Shoots (mg/g Air Dry Matter)	Roots (mg/g Air Dry Matter)
*Anchusa azurea*	Rosmarinic acid: 24	Allantoin: 0.21 Rosmarinic acid: 3.5
*Anchusa undulata*	Allantoin: 2.44 PHBA: 0.18 Rutin: 0.45 Rosmarinic acid: 25.9	Allantoin: 0.08 Rosmarinic acid: 27
*Borago officinalis*	Allantoin: 0.83 Rosmarinic acid: 3.7	Rosmarinic acid: 9.2
*Buglossoides purpureocaerulea*	Allantoin: 2.93 Rutin: 0.27 Rosmarinic acid: 33.1 Chlorogenic acid: 0.36	Allantoin: 1.86 Rosmarinic acid: 2
*Cerinthe minor*	Allantoin: 2.83 Hydrocaffeic acid: 1.57 Rosmarinic acid: 15.9	Allantoin: 0.21 Rosmarinic acid: 4.9
*Cynoglossum creticum*	Allantoin: 0.67 Rutin: 0.57 Hydrocaffeic acid: 0.90 Rosmarinic acid: 9.6	Allantoin: 0.66 Hydrocaffeic acid: 0.126 Rosmarinic acid: 2
*Echium italicum*	Allantoin: 9.59 PHBA: 0.20 Hydrocaffeic acid: 0.13 Rosmarinic acid: 3.3	Allantoin: 34.89 Hydrocaffeic acid: 0.13 Rosmarinic acid: 2.3 Shikonin: 0.07
*Echium russicum*	Allantoin: 7.41 Rutin: 0.47 Rosmarinic acid: 5.1	Allantoin: 10.85 Rosmarinic acid: 8.6 Shikonin: 0.16
*Echium vulgare*	Allantoin: 0.91 Rutin: 0.32 Hydrocaffeic acid: 0.82 Rosmarinic acid: 3.4 Chlorogenic acid: 0.36	Allantoin: 0.61 Hydrocaffeic acid: 0.08 Rosmarinic acid: 1.3 Shikonin: 0.28
*Lindelofia macrostyla*	Allantoin: 11.81 PHBA: 0.18 Rutin: 0.37 Hydrocaffeic acid: 0.68 Rosmarinic acid: 7.7	Allantoin: 11.98 Rosmarinic acid: 9.8
*Lithospermum officinale*	Allantoin: 2.36 PHBA: 0.17 Rutin: 0.75 Hydrocaffeic acid: 0.21 Rosmarinic acid: 1.2 Chlorogenic acid: 1.03	Allantoin: 0.81 Hydrocaffeic acid: 0.13 Rosmarinic acid: 1.8 Shikonin: 0.07
*Nonea lutea*	Allantoin: 0.99 Hydrocaffeic acid: 0.21 Rosmarinic acid: 1.2 Chlorogenic acid: 1.03	Allantoin: 0.60 Hydrocaffeic acid: 0.13 Rosmarinic acid: 1.8
*Pulmonaria mollis*	Allantoin: 1.04 Rosmarinic acid: 36.6	Allantoin: 9.75 Rosmarinic acid: 7.5
*Pulmonaria obscura*	Allantoin: 2.37 PHBA: 0.17 Rosmarinic acid: 15.6 Chlorogenic acid: 0.45	Allantoin: 13.14 Rosmarinic acid: 7.9
*Omphalodes verna*	Allantoin: 1.84 Rutin: 0.53 Rosmarinic acid: 12.9 Chlorogenic acid: 0.67	Allantoin: 13.14 Rosmarinic acid: 7.9
*Symphytum cordatum*	Allantoin: 0.63 Rosmarinic acid: 12.4	Allantoin: 13.14 Rosmarinic acid: 7.9
*Symphytum officinale*	Allantoin: 9.38 PHBA: 0.19 Hydrocaffeic acid: 0.20 Rosmarinic acid: 4.5 Chlorogenic acid: 0.62	Allantoin: 25.77Rosmarinic acid: 7.1

**Table 3:** Summary of the base material, methodology, food products monitored, and antioxidant activity of the shikonin-loaded biodegradable films from the literature.

Plant Species	Base Material	Shikonin Incorporation Method	Food Product Monitored	Antioxidant Activity Reported		Reference
				DPPH (%)	ABTS (%)	
*Lithospermum erythrorhizon*	TEMPO-oxidised cellulose nanofibres (CNFs)	10 wt% of the polymer added directly to the polymer–glycerol solution.	-	36%	84%	[127]
*Arnebia euchroma*	Softwood pulp cellulose	Regenerated cellulose films directly immersed in the ethanol extracts of *Arnebia euchroma* for 2, 4, 8, 12. and 24 h.	Fresh shrimp and pork	-	-	[122]
*Lithospermum erythrorhizon*	Carboxymethyl cellulose (CMC) and agar	10 wt% of the polymer added directly to the polymer–glycerol solution.	-	16%	40%	[128]
*Lithospermum erythrorhizon*	Ashless filter paper	Direct dipping of base material in 1 mg/mL shikonin (in ethanol) for 1 h.	Kimchi	14%	37%	[129]
Pure component shikonin	Hydroxypropyltrimethyl ammonium chloride chitosan	1 wt% and 2 wt% (of the polymer) shikonin added directly to the polymer solution.	Fresh shrimp	1%: 39% 2%: 62.74%	1%: 56% 2%: 88.57%	[123]
*Lithospermum erythrorhizon*	CMC-CNF	1 wt% (of the polymer) shikonin added directly to the polymer solution.	Fish	CMC film: 13.7% CNF film: 33.3%	CMC film: 93.5% CNF film: 57%	[124]
*Lithospermum erythrorhizon*	Gelatin–carrageenan	10 wt% of the polymer added directly to the polymer–glycerol solution.	Milk	38%	67%	[126]
*Lithospermum erythrorhizon*	CMC–agar with cellulose nanocrystals (CNCs)	10 wt% of the polymer added directly to the polymer–glycerol solution.	-	CMC–agar film: 12% CMC–agar–CNC: 18%	CMC–agar film: 72% CMC–agar–CNC: 81%	[130]
*Lithospermum erythrorhizon*	Starch–agar	40 mg shikonin in 5 mL ethanol added directly to the polymer solution.	Fresh shrimp	Starch film: 26.5% Agar film: 42.6%	Starch film: 66% Agar film: 83%	[125]
*Lithospermum erythrorhizon*	Gelatin–CNF	10 wt% of the polymer added directly to the polymer–glycerol solution.	Fresh shrimp	50%	60%	[131]
*Lithospermum erythrorhizon*	Regenerated cellulose from microcrystalline cellulose (MCC), natural hemp fibre, and cotton fibre	5% shikonin added directly to the polymer mixtures.	Fresh shrimp	MCC film: 37% Hemp fibre film: 35% Cotton fibre film: 33%	-	[132]

## Data Availability

No new data were created or analyzed in this study. Data sharing is not applicable to this article.
